# A Spectroscopic Approach to Investigate the Molecular Interactions between the Newly Approved Irreversible ErbB blocker "Afatinib" and Bovine Serum Albumin

**DOI:** 10.1371/journal.pone.0146297

**Published:** 2016-01-11

**Authors:** Amer M. Alanazi, Ali Saber Abdelhameed

**Affiliations:** Department of Pharmaceutical Chemistry, College of Pharmacy, King Saud University, P.O. Box 2457, Riyadh, 11451, Saudi Arabia; Islamic Azad University-Mashhad Branch, Mashhad, Iran, ISLAMIC REPUBLIC OF IRAN

## Abstract

The interaction of afatinib (AFB) with bovine serum albumin (BSA) was examined *via* fluorescence and UV-Vis spectroscopy. Spectrofluorimetric measurements revealed that AFB can strongly quench the BSA intrinsic fluorescence through producing a non-fluorescent complex. This quenching mechanism was thoroughly investigated with regard to the type of quenching, binding constant, number of binding locations and the fundamental thermodynamic parameters. Subsequently, the association constant of AFB with BSA was computed at three different temperatures and was found to range from 7.34 to 13.19 x10^5^ L mol^-1^. Thermodynamic parameters calculations demonstrated a positive Δ*S*^*Ɵ*^value with both negative Δ*H*^*ϴ*^and Δ*G*^*ϴ*^values for AFB–BSA complex, which in turn infers thata spontaneous binding is taking place with both electrostatic bonding and hydrophobic interactions participating in the binding of AFB and BSA. Similarly, the UV absorption spectra of AFB-BSA system were studied and confirmed the interaction. Conformational alteration of the protein upon binding to AFB was elaborated with the aid of three dimensional fluorescence measurements as well as synchronous fluorescence spectra.

## Introduction

**Fig 1:**
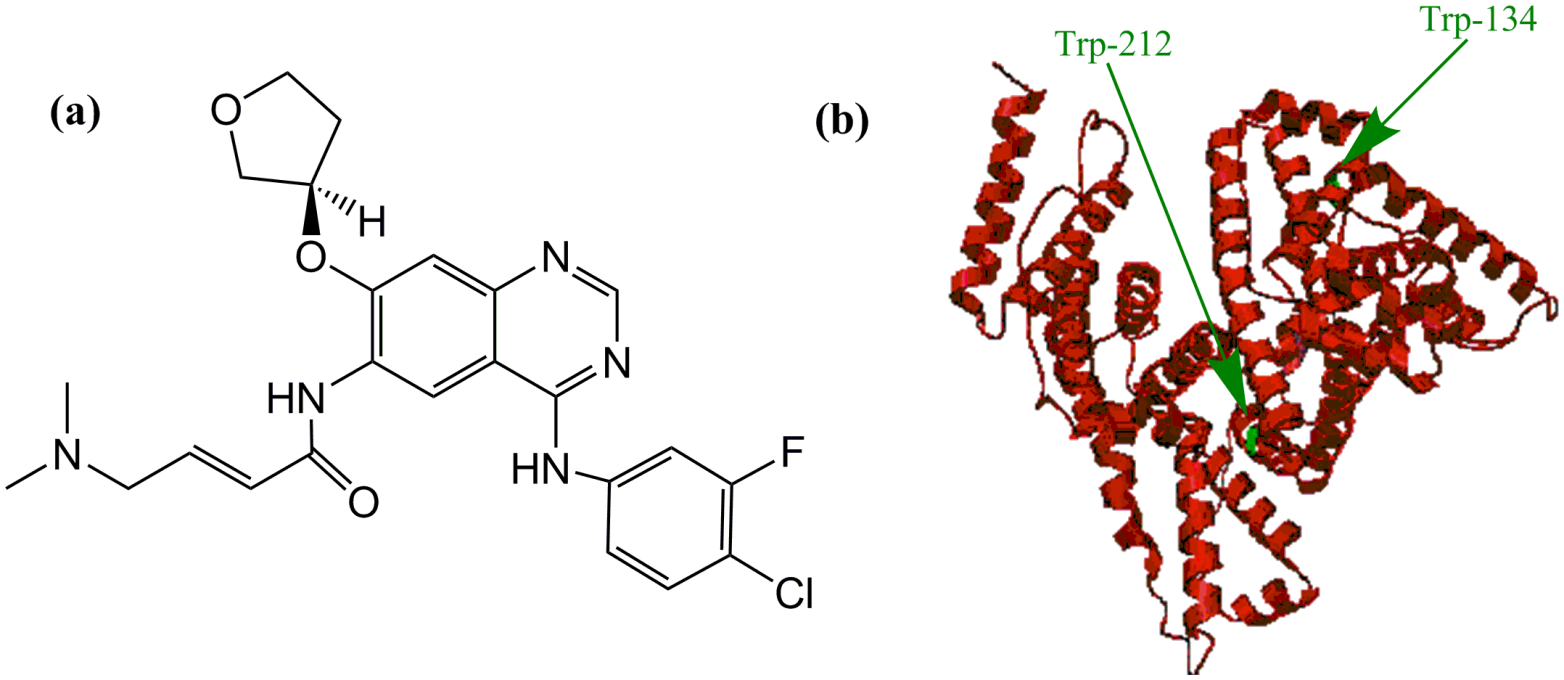
Chemical structure of AFB (a) and ribbon representation of BSA (b).

Afatinib (AFB; formerly known as BIBW 2992) (Fig 1), is an orally administered selective irreversible ErbB family blocker (EGFR/ErbB1,HER2/ErbB2 and ErbB4) with has wide-spectrum pre-clinical efficacy against EGFR mutations [1]. Due to the fact that, these receptors are essential in cellular proliferation and apoptosis, their inhibition by AFB may prevent tumor growth and spread. AFB is now marketed as Gilotrif^®^ tablets (Boehringer-Ingelheim pharmaceuticals, Inc. USA) in the form of afatinib di-maleate salt equivalent to 20, 30, and 40 mg afatinib base. AFB was granted the FDA approval for the management of patients with metastatic or locally advanced non-small cell lung cancer (NSCLC) with their tumors possess epidermal growth factor receptor (EGFR) exon 21 (L858R) substitution mutations or exon 19 deletions [2]. Therefore and with the advancement of drug development in cancer management, various possible interactions should be taken into account. Concomitantly, serum albumins, the multi-functional depots and transport carriers, are considered the predominant circulatory proteins in various organisms (50–60% of the total amount of plasma proteins) [3]. Serum albumins have been extensively studied in terms of their structural and physiological properties [4–6]. Their interaction with the different drugs may strongly influence the drugs’ apparent distribution volume as well as elimination rate. Therefore, studies on the drug-protein binding will aid in the interpretation of the metabolism and transport mechanism of the drug. An interesting member of the albumins family, is the bovine serum albumin (BSA) which also possess various physiological properties that involve its binding, conveying and delivery of a wide range of molecules [5,7–9]. Serum albumins are responsible for certain conformational dynamics and binding aggregation in solution [10]. BSA is structurally homologue to its human counterpart (HSA) with a well-studied structure [11–15]. Hence, the binding of the various drugs to BSA and serum albumins, in general, is an essential factor to determine the drug’s possible pharmacokinetic and pharmacodynamics profile. Several earlier investigations have reported the BSA-drugs interaction using spectroscopic tools [10,16–18] including recent studies of other members of the tyrosine kinase family [19,20]. In the present study, the intrinsic fluorescence of BSA quenched by AFB was investigated by the selective excitation of BSA’s tryptophan residues present at positions 134 and 212 of the 583 amino acids polypeptide chain [21–23] (Fig 1b). Thorough literature survey revealed that no single study was reported for investigating AFB and serum albumin binding. Therefore, the current work is sought to investigate the interaction of BSA and AFB utilizing fluorescence spectroscopy to provide an insight into their molecular interaction.

## Materials and Methods

### Materials

Unless otherwise stated all chemicals, reagents and solvents were of analytical grade and procured from BDH laboratory supplies (Poole, UK). Afatinib reference standard was acquired from Weihua Pharma Co. Ltd (Zhejiang, China). Techno Pharmchem (Haryana, India) supplied the bovine serum albumin (BSA). Ultrapure water of 18.2 MΩ was produced from a Millipore Milli-Q^®^ UF-Plus purification system (Millipore, MA, USA).

### Instruments and conditions

Fluorescence measurements were carried out in a 1-cm quartz cell with slits of 5nm (for excitation and emission) and *λ*_ex_ = 280 and monitoring at *λ*_em_ = 338 using a Jasco FP-8200 (Jasco International Co. Ltd. Tokyo, Japan). The AFB quenching effect on BSA was investigated at its maximum emission range (334–344 nm). All UV-Vis absorbance spectral determinations were performed on a Nanodrop^™^ 2000 UV-Vis spectrophotometer (Thermo Scientific, Wilmington, DE, USA). Experimental solutions were all made in1X phosphate buffered saline (PBS buffer) pH 7.4, with the pH recorded using an Adwa AD1030 pH-meter (ADWA Instruments Inc., Romania).

### Sample preparation

All solutions were prepared at room temperature, and were kept at −20°C. AFB was dissolved in methanol preparing a stock solution of 1.0 mgmL^-1^. Further, AFB solution was diluted (1.0 mL of stock solution) by 10 ml with methanol to yield a 100 μgmL^-1^ solution. Subsequent dilution of the latter solution was carried out using PBS buffer pH 7.4 producing a working solution of 20 μgmL^-1^.

A 1.0 mgmL^-1^solution of bovine serum albumin (BSA) was prepared in PBS buffer pH 7.4 and kept in a cool, dark place. Subsequent dilutions of BSA stock solution were made in PBS buffer pH 7.4 yielding a 100 μgmL^-1^working solution.

### Protein Concentration determination

Protein concentration was determined from the specific extinction coefficient of A^1%^_280_~6.7 for BSA using Nanodrop^™^ 2000 UV-Vis spectrophotometer (Thermo Scientific, Wilmington, DE, USA). Prior to measurements, BSA samples were filtered through 0.45 μm syringe filters.

### AFB–protein interactions

Following several preliminary spectrofluorimetric measurements of AFB and BSA, an optimum concentration of 100 μgmL^-1^was chosen for BSA with the drug concentration varying in the range of 0.3–10 μgmL^-1^. Fluorescence spectra were recorded at three different temperatures of 288, 298 and 309 K over a scan range of λ_em_290–500 nm after being excited at λ_ex_280 nm. To reduce the inner filter effect, both intensities of the fluorescence arising from excitation light absorption and emission light re-absorption were corrected using eq (1)[24,25]

$$F_{cor=F_{obs}\times e^{\left(A_{ex}+A_{em}\right)/2}}$$ (1)

Here, the corrected and observed fluorescence intensities are represented as *F*_*cor*_ and *F*_*obs*_, respectively. While, values of the AFB absorbance at excitation and emission wavelengths are represented as *A*_*ex*_ and *A*_*em*_, respectively.

### UV-Vis spectrophotometric determinations

UV-Vis absorption determinations of BSA in the presence and absence of AFB were made in the range of 220–350 nm. BSA concentration was kept constant at 1.0 mgmL^-1^with using AFB concentration of 5.0and 10.0 μgmL^-1^ for binding measurements and 1.0 μgmL^-1^ for AFB reference measurement.

## Results and Discussion

### Fluorescence Measurements

**Fig 2:**
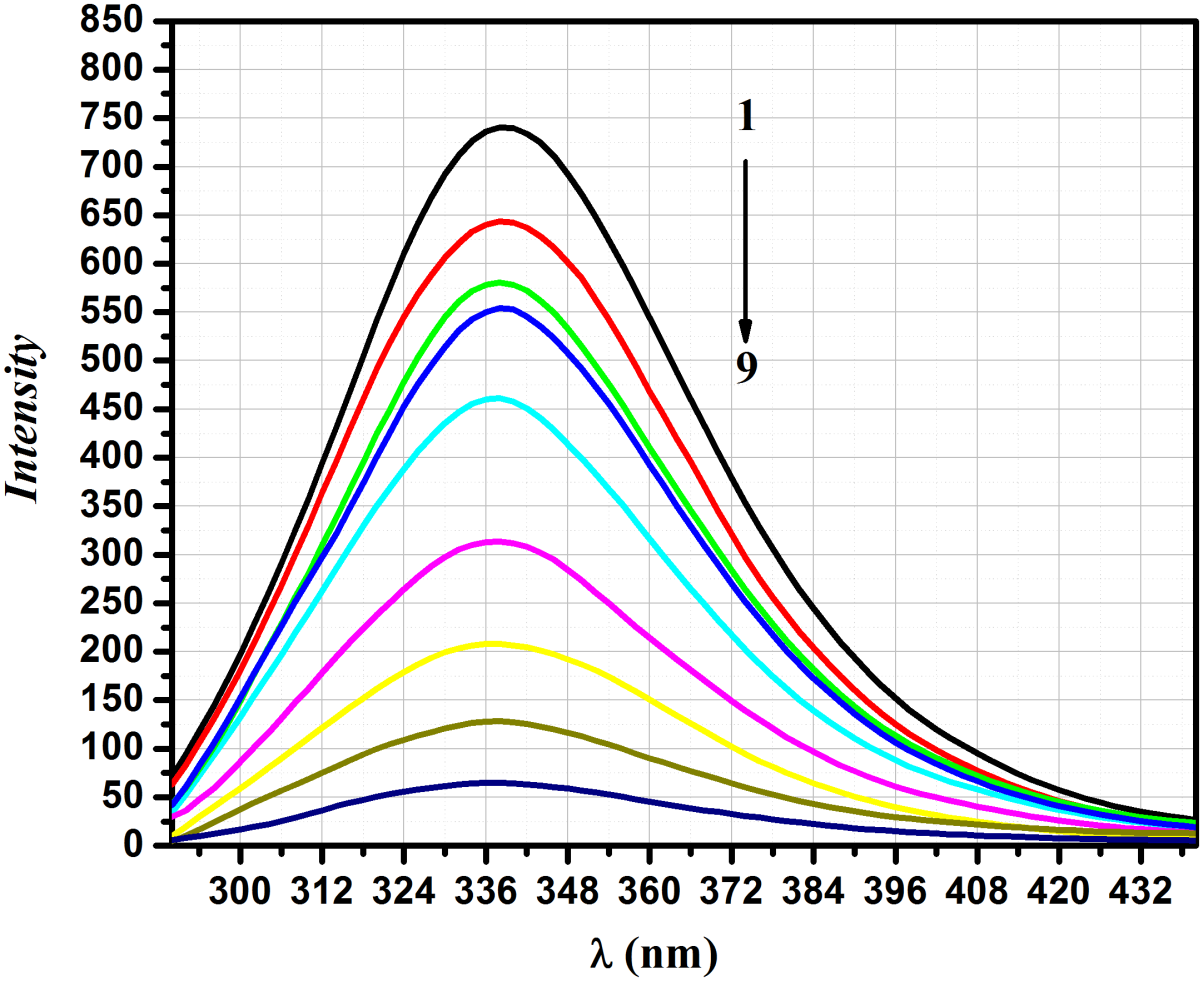
Emission spectra of BSA (100 μg.mL^-1^) only (1) and following BSA binding to AFB at a concentration series of 0.3 μ g.mL^-1^ (2), 0.4 μg.mL^-1^ (3), 0.5 μg.mL^-1^ (4), 1.0 μ g.mL^-1^ (5), 3.0 μg.mL^-1^ (6), 5 μg.mL^-1^ (7), 7.0 μg.mL^-1^ (8) and 10 μg.mL^-1^(9).

Fluorescence measurements among a variety of spectroscopic tools can yield plenty of information of the binding of proteins and/or small molecules to proteins, such as the binding mechanism, type, binding parameters, conformational changes, *etc*. [26–28]. Quenching of the fluorescence indicates reduction of the fluorescence intensity of a given fluorophore *via* different molecular interactions [29,30]. The fluorescence spectra of BSA in absence and presence of a concentration series of different AFB were determined in the range of λ_em_290–500 nm following excitation at λ_ex_ 280 nm. AFB resulted in quenching of the BSA fluorescence intensity in a linear manner (Fig 2) without affecting the BSA emission wavelength and shape of the peak. The observed results reflect a complex formation between AFB and BSA [31].

### Fluorescence quenching mechanism

Fluorescence quenching mechanisms are categorized into dynamic quenching and staticquenching [12,32]. Dynamic quenching is a result of diffusion whereas static type of quenching is basically due to formation of a ground state complex. Moreover, both types have different temperature dependence *viz*. quenching constants are supposed to increase at higher temperatures in dynamic quenching. Conversely, a diminished stability and lower quenching constants are favored with elevated temperature in static quenching. Quenching results were interpreted using the Stern–Volmer (Eq 2) [33] and Lineweaver–Burk mathematical formulas (Eq 3) [34], both equations have been commonly reported to describe dynamic and static quenching.

F0F=1+KSVCQ=1+Kqτ0CQ (2)

$${\left(F_{0}-F\right)}^{-1}=\;F_{0}{}^{-1}+K_{LB}{}^{-1}F_{0}{}^{-1}C_{Q}{}^{-1}$$ (3)

**Fig 3:**
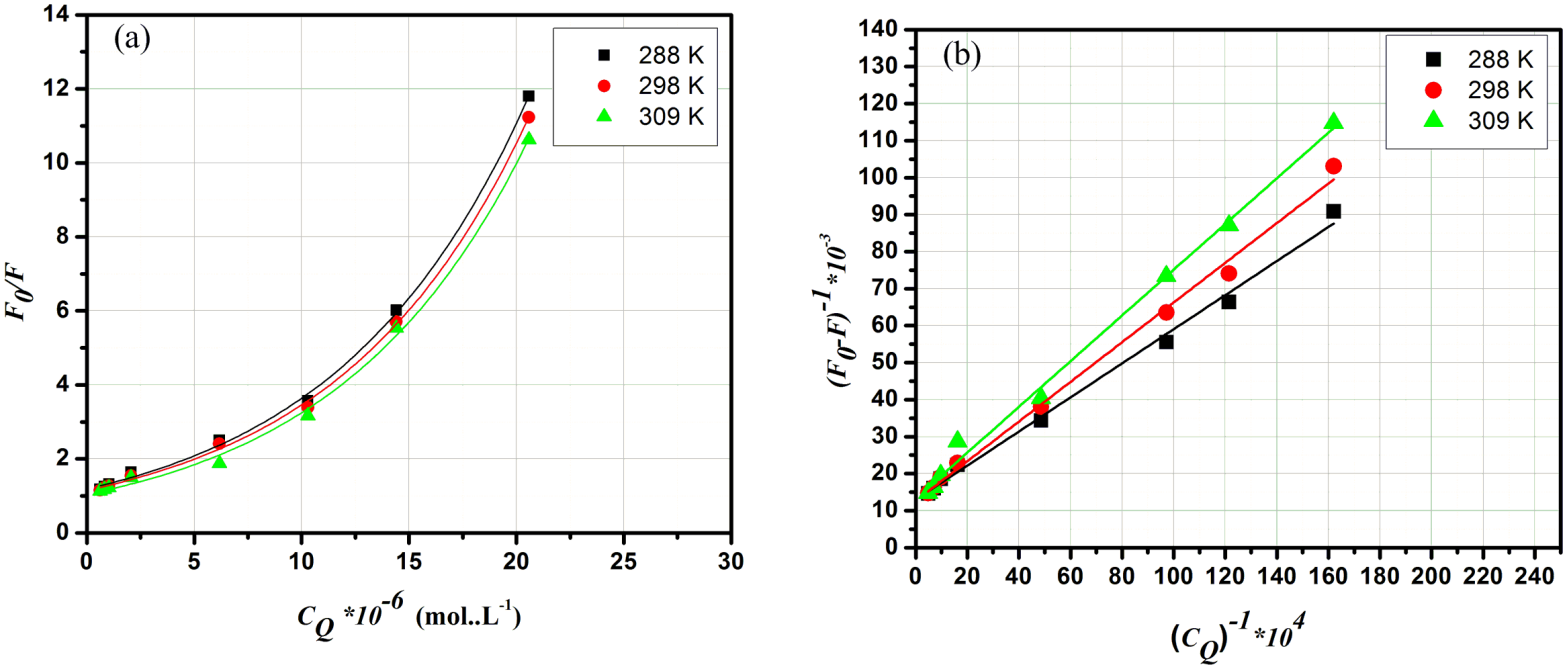
Stern–Volmer (a) and Lineweaver–Burk (b) plots at various temperatures.

Where *F*_0_ and *F* the fluorescence intensities of BSA without and with the addition of AFB, respectively, *C*_*Q*_ the concentration of AFB (quencher) and, *K*_*SV*_, *K*_*LB*_ are the Stern–Volmer and Lineweaver–Burk constants, respectively. While, the quenching rate constant is *K*_q_and*τ*_0_ isthe mean protein lifetime in absence of the quencher. Previous reports have demonstrated that, over a defined range of concentration, if the quenching type is single static or dynamic quenching then the curve of *F*_0_/*F* versus *C*_*Q*_(Stern–Volmer curve)would be linear [16]. Whilst, a linear curve of(*F*_0_−*F*)^−1^ versus *C*_Q_^-1^ (Lineweaver–Burk curve) is an indication of a static quenching [35]. On the other hand, an ascending curvature of the Stern–Volmer plot refers to combined(dynamic and static) quenching[36]. It can be observed from Fig 3a that the binding of AFB and BSA result in linear Stern–Volmer curves at lower AFB concentration(0.3–3.0 μg.mL^-1^) which become significantly upward bent at higher AFB concentrations(5.0–10.0 μg.mL^-1^). This supports the hypothesis of a single quenching (static or dynamic quenching) at lower AFB concentration, while a combined quenching (both static and dynamic) would be more appropriate at higher AFB concentrations. Additionally, Fig 3b of the Lineweaver–Burk plots(0.3–10.0 μg.mL^-1^) demonstrate that under the studied concentration range of AFB, the curves of (*F*_0_−*F*)^−1^*vs*.*C*_*Q*_^*-1*^ were linear that infers the presence of clear characteristics of a static quenching. Furthermore, the *K*_*SV*_ and *K*_*LB*_ values summarized in Table 1 are decreasing upon the steady increase in temperature that in turn is in a good agreement with the static quenching hypothesis [36].

**Table 1:** Parameters computed from both Stern-Volmer and Lineweaver–Burk relations for AFB-BSA binding.

Temperature (T)*(K*	Stern-Volmer parameters			Lineweaver–Burk parameters	
	*K*_*SV*_ x 10^5^(Lmol^−1^)	*K*_*q*_×10^13^(Lmol^−1^s^−1^)	*r*^*2*^	*K*_*LB*_ x 10^5^(Lmol^−1^)	*r*^*2*^
288	1.12±0.087	1.12	0.9986	2.82±0.12	0.9948
298	1.09±0.084	1.09	0.9987	2.35±0.11	0.9956
309	1.04±0.134	1.04	0.9970	2.15±0.12	0.9955

Moreover, the formation of a non-fluorescent complex was confirmed by the quenching rate constants, *K*_q_, values shown in Table 1, which were calculated using eq 4

Kq=KSVτ0 (4)

Where the value of *τ*_0_(average protein lifetime without the quencher),*τ*_0_of a biopolymer is 10^−8^*s*^*−1*^)[37]. The obtained *K*_q_ values are higher than the previously reported values for various quenchers with the biopolymer of 2×10^10^ LM^−1^s^−1^[37]. This in turn infers that the quenching in case of AFB-BSA is a result of a complex formation and not induced by dynamic collision [35].

### Binding mode and binding sites

Presuming independent binding of small molecules to a group of equivalent sites on a macromolecule, hence, eq 5 can explain the equilibrium between free and bound molecules [36,38]:

$$\text{log}\left(\frac{F_{0}-F}{F}\right)=\text{log}K+n\text{log}C_{Q}$$ (5)

In this equation (Eq 5)the binding constant is referred to as *K*while, binding sitesnumber on a BSA molecule is *n*. Plotting log(*F*_*0*_*−F)/F vs*. log*C*_*Q*_ (S1 Fig) could yield *K* and *n* values which are summarized in Table 2 at the investigated temperatures. These values demonstrate a reduction in the binding constant and to a lesser extent the *n* value with the increase in temperature, producing a less stable afatinib–BSA complex. Furthermore, *n* values were found to be nearly ~1 that infers the existence of one association site between BSA and afatinib.

**Table 2:** Summary of the thermodynamic parameters for AFB-BSA interaction along with binding parameters *K* and *n*.

Temperature(T) *(K)*	Δ*G*^*θ*^ (kJmol^−1^)	Δ*H*^*θ*^(kJmol^−1^)	Δ*S*^*θ*^(Jmol^−1^K^−1^)	*K* × 10^5^(Lmol^−1^)	n*	*r*^*2*^
288	-33.75±0.04	-20.69±0.62	45.34±2.85	13.19±0.14	1.13±0.01	0.9787
298	-34.19±0.07			9.87±0.46	1.09±0.01	0.9771
309	-34.69±0.12			7.34±0.31	1.07±0.01	0.9745

* All values are average of three determinations

### Thermodynamics parameters and nature of the binding forces

Thermodynamic variants namely, enthalpy (Δ*H*^*ϴ*^) and entropy (Δ*S*^*ϴ*^) of AFB–BSA interaction are significant to confirm the binding forces. The thermodynamic process is deemed responsible for formation of the complex as the binding constant depends on primarily on temperature. In general, the interaction of a ligand and a macromolecule may involve one or more of the different binding forces *viz* van der Waals, hydrophobic, electrostatic forces and/or formation of hydrogen bonds. Previous reports including our group’s findings on the sign and magnitude of the different thermodynamic parameter associated with the various types of protein-ligand interactions [39–44] concluded that, a hydrophobic interaction is consistent with positive Δ*H*^*θ*^ and Δ*S*^*θ*^ of a system, while hydrogen bonding and van der Waals forces result in negative Δ*H*^*θ*^and Δ*S*^*θ*^ values. Additionally, involvement of the electrostatic forces usually renders a negative Δ*H*^*θ*^ and a positive Δ*S*^*θ*^[39–44]. In the present study, thermodynamic parameters of the AFB-BSA system were computed using eqs 6 and 7:

$$\text{ln}K=\frac{-\Delta H^{\theta }}{RT}+\frac{\Delta S^{\theta }}{R}$$ (6)

$$\Delta G^{\theta }=\Delta H^{\theta }-T.\Delta S^{\theta }$$ (7)

**Fig 4:**
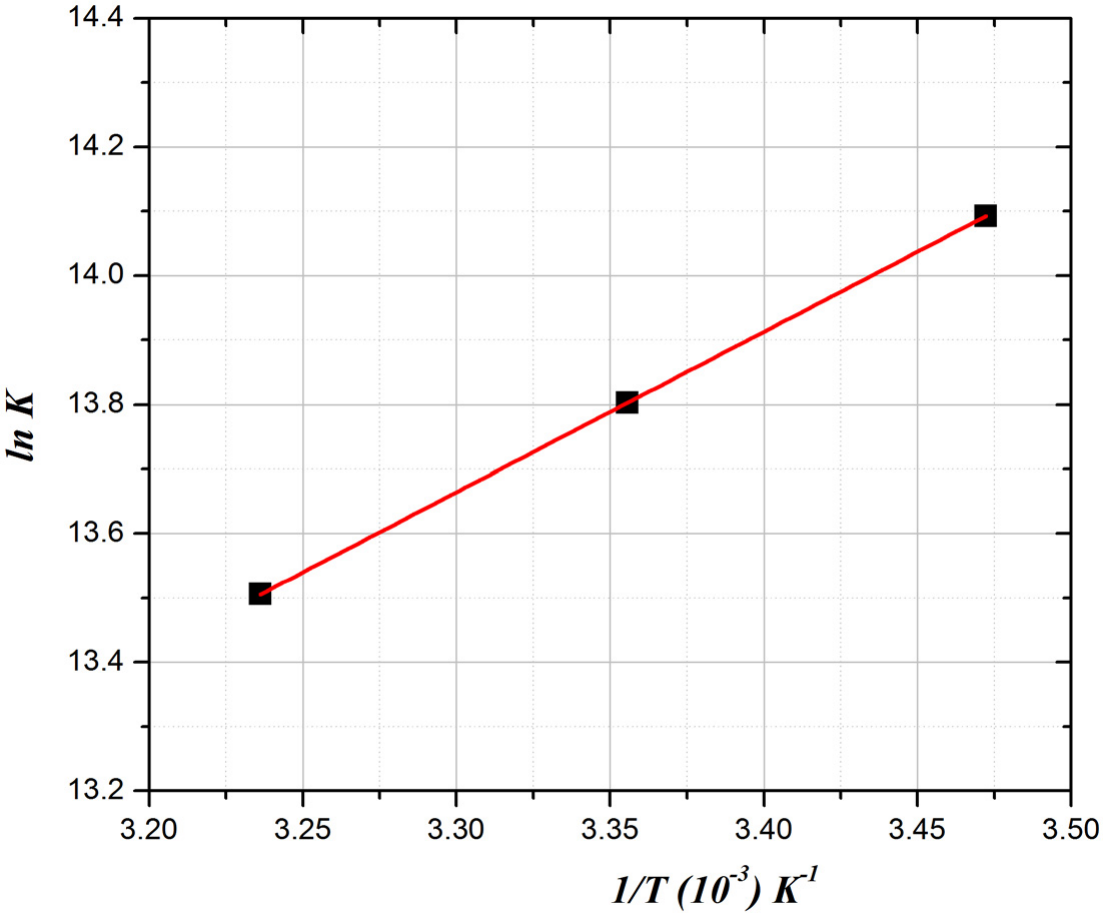
A representative Van’t Hoff plot for AFB-BSA binding.

In these equations, binding constant is referred to with the letter *K* while *R* stands for the gas constant, while *T* is temperature (in Kelvins) and Δ*G*^*θ*^is the free energy change. Hence, plotting the values of *lnK* on the *y*-axis and *1/T*on the *x*-axis (Fig 4) will result in the calculation of Δ*H*^*θ*^, Δ*G*^*θ*^ and Δ*S*^*θ*^.

Based on the previously mentioned rules for the main binding forces, the results summarized in Table 2 reveal that the AFB-BSA binding can not be accounted for as a single intermolecular force model. As from the water structure point of view, a positive Δ*S*^*θ*^ value is usually considered an evidence for hydrophobic interaction [38,44]. Additionally, for AFB-BSA system under our experimental pH of 7.4, AFB is over 96% ionized based on its predicted pKa value (8.81 due to the dimethylamine moiety); along with the obtained negative Δ*H*^*θ*^ and positive Δ*S*^*θ*^, so electrostatic interaction cannot be excluded from the binding. Hence, it is probably that hydrophobic and electrostatic forces are involved in this binding process

### UV–vis absorption spectra

**Fig 5:**
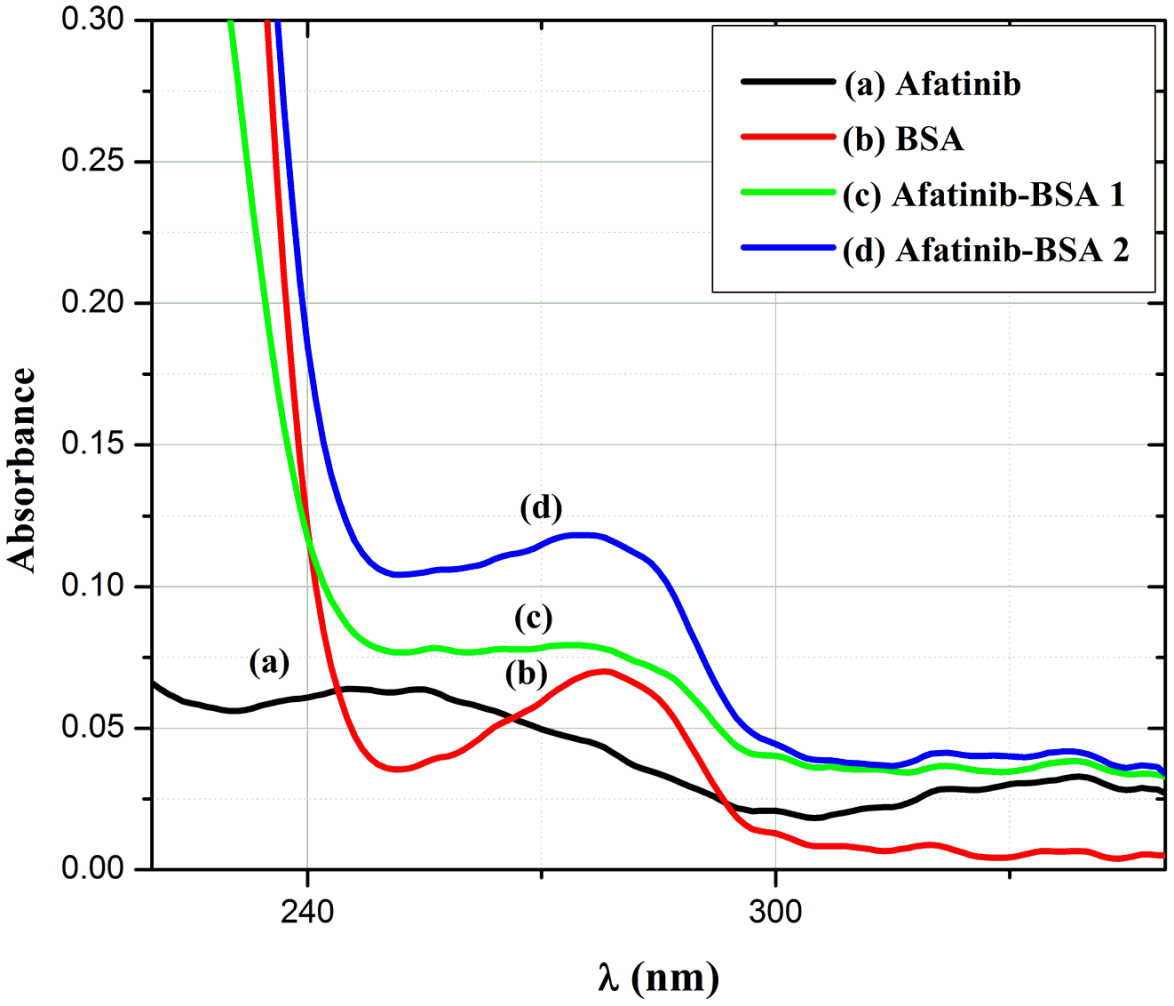
UV spectra of (a) 1.0 μgmL^-1^AFB (b) 1.0 mgmL^-1^ BSA (c) AFB (5.0 μgmL^-1^)+BSA(1.0 mgmL^-1^) (d) AFB (10.0 μgmL^-1^)+BSA(1.0 mgmL^-1^).

Fig 5 shows the measured absorption spectra for AFB and BSA and proves the formation of a complex between AFB and BSA. Moreover, the UV absorption intensity enhanced gradually for BSA following addition of AFB concentrations, which may infer the extension of the BSA peptide strands with the AFB gradual increment.

### Effect of AFB on BSA Conformation

#### Synchronous fluorescence

**Fig 6:**
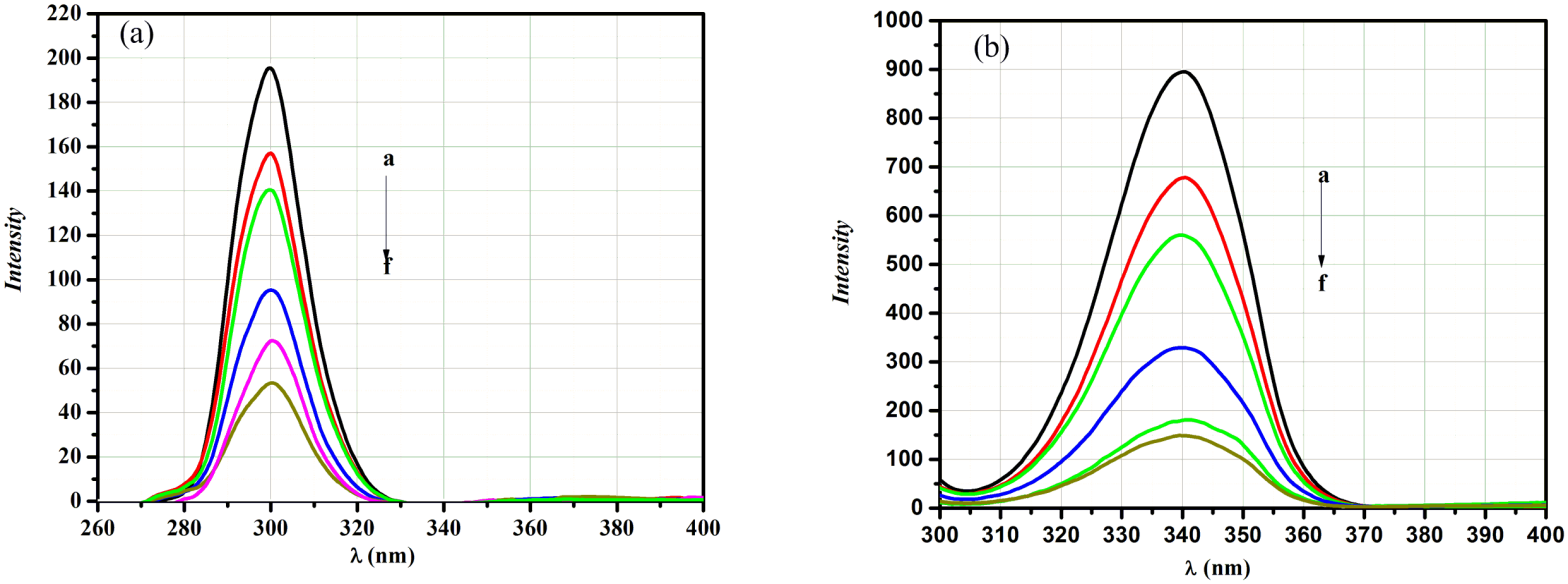
Spectra of the synchronous fluorescence of BSA (100 μg.mL^-1^) with the addition of AFB (a-f) = (0, 0.5, 1.0, 3.0, 5.0 and 10.0 μg.mL^-1^) at (a) Δλ = 15 nm (b) Δλ = 60 nm.

Synchronous fluorescence spectra of BSA indicates the fluorescence of tyrosine (Tyr) and tryptophan residues (Trp.) of BSA at wavelength intervals of Δλis 15 nm and 60 nm, respectively[45,46]. It can be seen from Fig 6 that the intensity of the emission spectra of Tyr. and Trp. was diminished with no clear peak shift. Comparing the quenching effect of AFB on the fluorescence intensity of Tyr. and Trp. residues, it is clear that a Trp. Quenching is more significant, which may refer to that the binding site of AFB nearer to tryptophan residues.

#### Three dimensional fluorescence measurements

**Fig 7:**
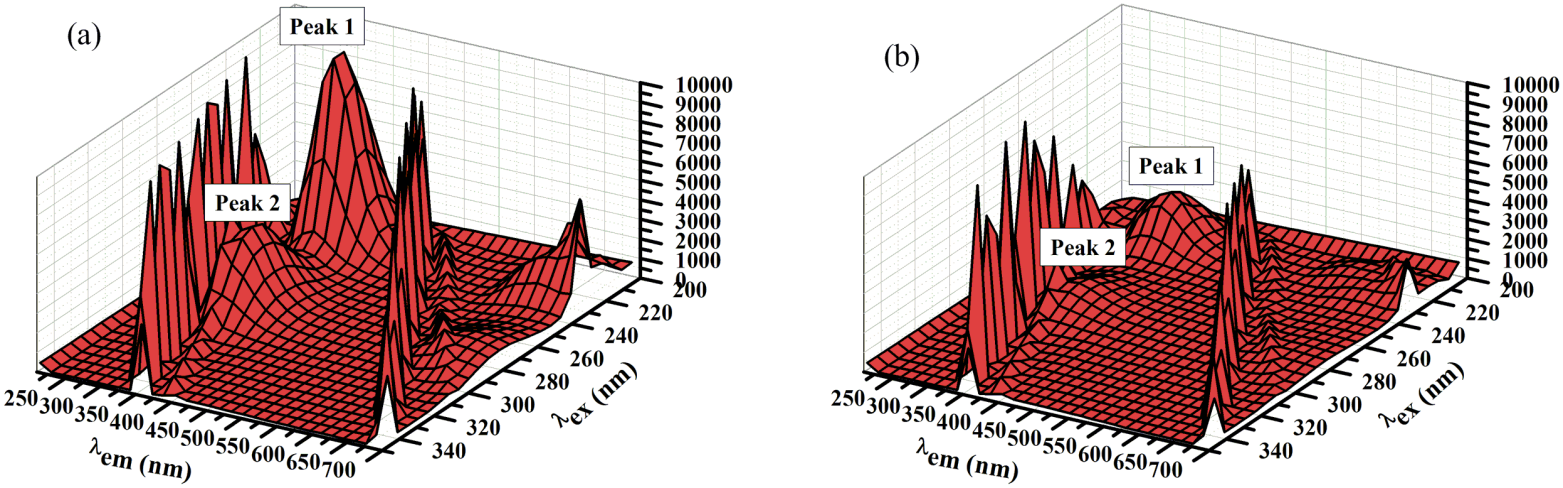
3D spectra of BSA (100 μg.mL^-1^) in (a) absence and (b) presence of AFB (5 μg.mL^-1^).

**Fig 8:**
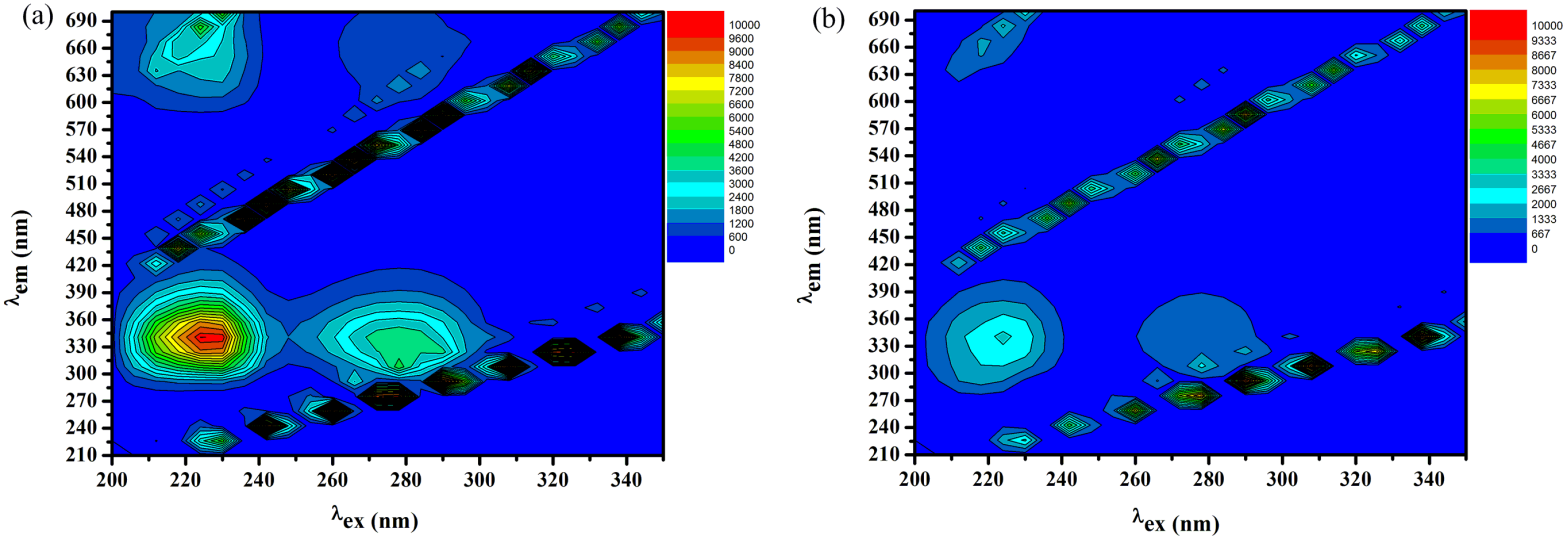
Contour plot of the fluorescence intensity spectra of BSA (a) AFB-BSA system (b).

Measurements of the three-dimensional (3D) fluorescence were performed with the calculations of different characteristic 3D parameters are reported in Table 3. Figs 7 and 8 show that BSA possess different fluorescence peaks, where, peak 1, (λ_ex_224→λ_em_336) which essentially refers to the fluorescence feature of *n*→π* transition of the polypeptide backbone of BSA structure, C = O[47]. While, peak 2, (λ_ex_278→λ_em_334) infers the spectral attributes of Trp. and Tyr. residues[18]. With its binding to AFB the BSA fluorescence peaks were quenched as evidenced from Figs 7b and 8b.

**Table 3:** Parameters of the 3D fluorescence for AFB-BSA binding.

	BSA		AFB-BSA	
	1^st^ Peak	2^nd^ Peak	1^st^ Peak	2^nd^ Peak
**Position of the peak (*λ*ex/*λ*em, nm/nm)**	224/336	278/334	224/336	278/334
**Relative intensity (*RFI*)**	9993.22	4258.31	2845.65	1290.87
**Stokes shift Δ*λ/*nm**	112	56	112	52

## Conclusions

The current study provided an approach for investigating the interactions of the new tyrosine kinase inhibitor, afatinib (AFB), with BSA for the first time using fluorescence-quenching technique. AFB was shown to quench the fluorescence of BSA *via* static type of binding and formation of a non-fluorescent complex. Binding constant for AFB-BSA complex was computed to be in the order of 10^5^ Lmol^−1^. The calculated thermodynamic parameters were consistent with the rule of Δ*G*^*θ*^< 0; Δ*H*^*θ*^<0; Δ*S*^*θ*^> 0 which mainly infer a spontaneous interaction that may involve both hydrophobic and electrostatic binding forces. Since serum albumins are known to have diverse functions, particularly as carrier molecules for several drugs. The work presented in this study can form an important tool in assessing the pharmacological properties of AFB when used in cancer patients.

## Supporting Information

S1 Fig Binding mode and binding sites. Plots of log[(*F0*-*F*)/*F*] *vs*. log[*C*_*Q*_] for AFB–BSA interaction at different temperatures.
(PDF)
