# Supplementary material for: A Spectroscopic Approach to Investigate the Molecular Interactions between the Newly Approved Irreversible ErbB blocker "Afatinib" and Bovine Serum Albumin
Source: PLoS One. 2016 Jan 11;11(1):e0146297. doi: 10.1371/journal.pone.0146297 (PMC4709191; doi:10.1371/journal.pone.0146297)
Supplement: S1 Fig — Plots of log[(F0-F)/F] vs. log[CQ] for AFB–BSA interaction at different temperatures. (PDF) [file pone.0146297.s001.pdf]

Binding mode and binding sites

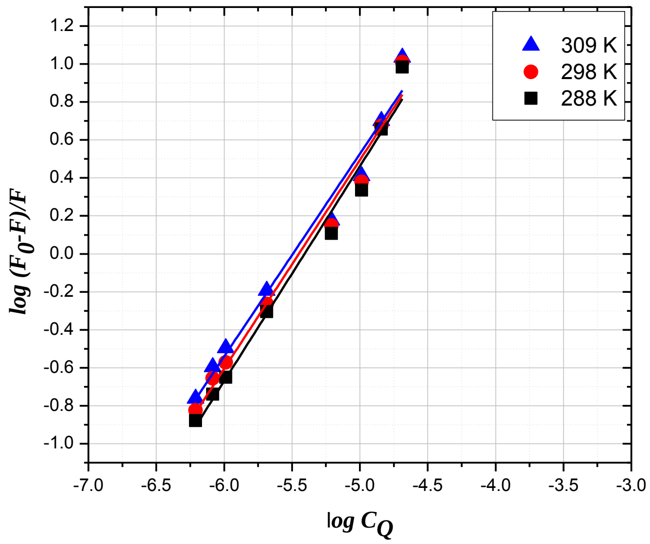

S1 Fig: Plots of log[(*F0-F*)/*F*] *vs.* log[C_Q] for AFB–BSA interaction at different temperatures
